# Nutrient-Deprived Retinal Progenitors Proliferate in Response to Hypoxia: Interaction of the HIF-1 and mTOR Pathway

**DOI:** 10.3390/jdb4020017

**Published:** 2016-05-19

**Authors:** Helena Khaliullina, Nicola K. Love, William A. Harris

**Affiliations:** Department of Physiology, Development and Neuroscience, University of Cambridge, Cambridge CB2 3DY, UK; nicolakatelove@gmail.com

**Keywords:** neural progenitors, hypoxia, nutrition, proliferation

## Abstract

At a cellular level, nutrients are sensed by the mechanistic Target of Rapamycin (mTOR). The response of cells to hypoxia is regulated via action of the oxygen sensor Hypoxia-Inducible Factor 1 (HIF-1). During development, injury and disease, tissues might face conditions of both low nutrient supply and low oxygen, yet it is not clear how cells adapt to both nutrient restriction and hypoxia, or how mTOR and HIF-1 interact in such conditions. Here we explore this question *in vivo* with respect to cell proliferation using the ciliary marginal zone (CMZ) of *Xenopus*. We found that both nutrient-deprivation and hypoxia cause retinal progenitors to decrease their proliferation, yet when nutrient-deprived progenitors are exposed to hypoxia there is an unexpected rise in cell proliferation. This increase, mediated by HIF-1 signalling, is dependent on glutaminolysis and reactivation of the mTOR pathway. We discuss how these findings in non-transformed tissue may also shed light on the ability of cancer cells in poorly vascularised solid tumours to proliferate.

## 1. Introduction

It is well known that normally cells depend on nutrients for growth. Indeed, nutrient deprivation (ND) blocks proliferation in many *in vitro* systems through a check-point in the cell cycle [1,2,3,4]. We have previously shown that, *in vivo*, cellular proliferation in the ciliary marginal zone (CMZ), the proliferating niche of the continuously growing retinas of lower vertebrates, is dependent on nutrition, and that mechanistic Target of Rapamycin (mTOR) acts as a restriction point regulator, such that in ND, the inactivation of mTOR blocks proliferation of early progenitors while allowing late progenitors to differentiate [5]. Interestingly, proliferating cells in the CMZ also show a Warburg metabolism, fuelling growth through glycolytic products and relying less on oxidative phosphorylation to meet their energy demands [6]. Cells that are exposed to hypoxia also show preference for glycolysis over oxidative phosphorylation. Many solid tumours lack vascularization, so they experience both ND and hypoxia. Intriguingly, hypoxia can induce a proliferative response in cancer cells [7,8], suggesting that these cells may be primed to respond in a different way to hypoxia than normal cells do. We therefore wondered how hypoxia affects the proliferation of nutrient deprived CMZ cells. 

The adaptive responses to starvation and hypoxia depend largely on two major sensory proteins, mTOR and Hypoxia-Inducible Factor 1 (HIF-1). mTOR is activated by growth factor receptors, amino acids and other environmental stimuli that influence overall energy levels in tissues [9,10,11,12,13,14]. The activity of mTOR stimulates growth and proliferation. In the CMZ of *Xenopus*, nutrient deprivation inhibits the mTOR pathway leading to the cessation of cell proliferation that is normally continuously underway in the CMZ of the *Xenopus* retina [5]. HIF-1 activity is subject to tight regulation by oxygen concentration [15,16,17,18,19]. Hypoxia is also known to inhibit the cell cycle in non-transformed cells [20]. Some interactions between the HIF-1 pathway and the mTOR pathway have been described. For instance, the translation of the HIF-1 protein can be regulated by mTOR signalling [21,22,23] which itself can be activated [24] or inhibited by HIF-1 [25,26,27]. Yet it is unclear from these studies how proliferating cells would respond when faced with both ND and hypoxia. To approach this question, we used the CMZ of *Xenopus*, a stem cell niche where there is continuous proliferation throughout postembryonic growth. The advantage of using this system is that the effects of nutrient deprivation and hypoxia can be studied individually and *in vivo* in whole living animals. Nutrient deprivation can be achieved by dissection of yolk, which does not interfere with survival of the embryo (see Materials and Methods). Hypoxia can be induced by placing entire embryos in a hypoxic chamber, and be undertaken on either nutrient-deprived or normal-fed embryos to investigate different combinations of conditions. Under low nutrient conditions, progenitor cells in the CMZ are known to cease proliferating due to the inhibition of the mTOR pathway [5]. Here we show that this phenomenon can be reversed under hypoxic conditions, with ND cells in the CMZ upregulating mTOR signalling and increasing their proliferation in response to low oxygen. Furthermore, we demonstrate that this response is mediated by HIF-1 signalling and depends on both glutaminolysis and the reactivation of the mTOR pathway.

## 2. Materials and Methods

### 2.1. Animal Maintenance and Pharmacological Treatment

*Xenopus laevis* embryos, obtained by *in vitro* fertilization, were raised in 0.1× Modified Barth’s solution (MBS) and staged according to [28]. For all experiments using nutrient-deprivation, except the glutamine re-feeding experiment using retinal explants, whole and alive embryos were used. Embryos were anaesthetized and nutrient deprived by yolk dissection at stage 35, and analysed at stage 38, after 24 h, as described previously [5]. Embryos were then maintained in 0.1× MBS throughout the experiment. In drug treatment conditions, 100 nM Echinomycin (Sigma-Aldrich, Gillingham, UK), 50 µM bis-2-(5-phenylacetamido-1,3,4-thiadiazol-2-yl)ethyl sulfide (BPTES) (Sigma) or 5 µM Rapamycin (Calbiochem/Merck-Millipore, Watford, UK) were bath-applied in 0.1× MBS.

### 2.2. Induction of Hypoxia

Whole and alive normal-fed and nutrient-deprived embryos at stage 38 were placed into a hypoxic bath chamber, which was maintained under a constant infusion of a mixture of 5% oxygen and 95% CO_2_, for 5 h. These embryos were used for the Western blot and immunostaining. For 5-ethynyl-2′-deoxyuridine (EdU) incorporation, the embryos were incubated in the hypoxic bath chamber for 3 h followed by incubation in bath-applied EdU in the same hypoxic chamber for another 2 h. For the time-course of EdU incorporation, normal-fed and nutrient-deprived embryos were placed into the hypoxic bath chamber for different times and subsequently given a 1 h pulse of EdU bath-applied in the hypoxic chamber. 

### 2.3. EdU Labelling

To mark cycling cells, 5 mM EdU was bath applied to embryos for 2 h prior to fixation. Embryos were fixed, sectioned and the EdU incorporation was detected on 14 µm sections using Click-iT chemistry kit performed in accordance with the manufacturer’s instructions (Molecular Probes, Thermo Fisher, Paisley, UK). Fluorescent sections were visualized under the confocal microscope and EdU-positive cells were counted blind. Statistical analysis was determined by two-tailed Student’s *t*-test (see Statistical Analysis).

### 2.4. Ex Vivo Cultures

An *ex vivo* culture method was adapted from that previously described by [29] and used only for the experiments with retinal explants. Embryos, nutrient-deprived by yolk dissection at stage 39, were grown for 24 h at 16 °C. Embryos were then washed in sterile 0.1× MBS with Penicillin/ Streptomycin/ Amphotericin (PSF), and anesthetized in MS222 solution. Retinas were removed under sterile conditions, washed and cultured at 20 °C on Parafilm to prevent adhesion, in 4 well or 35 mm culture dishes. For glutamine experiments, embryos were nutrient-deprived for 24 h at stage 36, grown to stage 41 and their retinas were then explanted and cultured overnight either in 60% L15 (Fisher Scientific, Loughborough UK) or 60% L15 without l-glutamine (Sigma) with/without substitution of 2.05 mM l-glutamine (Sigma).

### 2.5. Immunostaining

Immunostaining was performed on 14 μm sections using rabbit anti-Phospho Ribosomal protein S6 (Ser 235) (1:500, Cell Signaling) and Alexa Fluor 594 goat anti-rabbit (1:1000) (Invitrogen, Thermo Fisher, Paisley, UK) antibodies. Antigen retrieval was performed by steaming with 0.01 M Sodium Citrate, pH = 6, prior to staining with anti-pS6 antibody. Nuclei were labelled with 0.1 μg/mL 4′,6-diamidino-2-phenylindole (DAPI) (Invitrogen). Immunofluorescent sections were imaged with Laser Confocal Microscopy (Olympus Fluoview FV1000, Southend-on-Sea, UK). Sections examined, compared and depicted in all figures were all at the level of the optic nerve. All images were analysed using Volocity 6.3 (Perkin Elmer, Waltham, MA, USA).

### 2.6. Statistical Analysis

The number of independent experiments is indicated in each figure legend. For EdU quantification, a minimum of 8 cross-sections was used for analysis of each condition. Each examined section was at the level of optic nerve, therefore the number of examined sections corresponds to the number of examined retinas. Number of EdU-positive cells present in each section was counted blind. A two-tailed Student’s *t*-test was used to calculate the P values between two conditions. The two conditions that were compared are always indicated by two hooked ends of horizontal line connecting the conditions. Above the line, * indicates a *p* value < 0.05, ** indicates a *p* value < 0.01, and *** indicates a *p* value < 0.001. Error bars indicate *n* independent experiments. In each experiment, between 8 and 17 retinas were examined. 

### 2.7. Western Blot

Western blots were performed on retinal tissue lysates dissected from embryos treated as described, using at least 20 retinas per condition. Ten retinas for each condition were lysed on ice in RIPA buffer (Sigma) with 1:100 protease inhibitor (Sigma), homogenised by sonication and centrifuged. Supernatants were supplemented with SDS loading buffer and denatured at 80 °C for 5 min. Samples were run on precast 12% gels and transferred according to manufacturer’s protocol (Biorad, Hertfordshire, UK). For detection, rabbit anti-HIF-1 (1:500, ab2185 Abcam), rabbit anti-pS6 Ser235/236 (1:1000, Cell Signalling), mouse anti-α-tubulin (1:5000, Abcam), goat anti-rabbit HRP-conjugated (1:1000 Abcam) and goat anti-mouse HRP-conjugated (1:1000; Abcam) antibodies were used. Western blots were visualized using the ECL system (Amersham, Buckinghamshire, UK) and quantified in FiJi where, the pixel intensities of each band were quantified and normalized to the pixel intensity of the background. The error bars represent the standard deviations between the same conditions in different experiments, the number of experiments is stated in the corresponding figure legend. Experimental antibodies were normalised to loading controls and then experimental conditions (e.g., ND) normalised to normal-fed untreated controls to determine the ratio to control that was depicted.

## 3. Results

### 3.1. Hypoxia Regulates Proliferation in the CMZ Differently Dependent on Nutritional State

**Figure 1:**
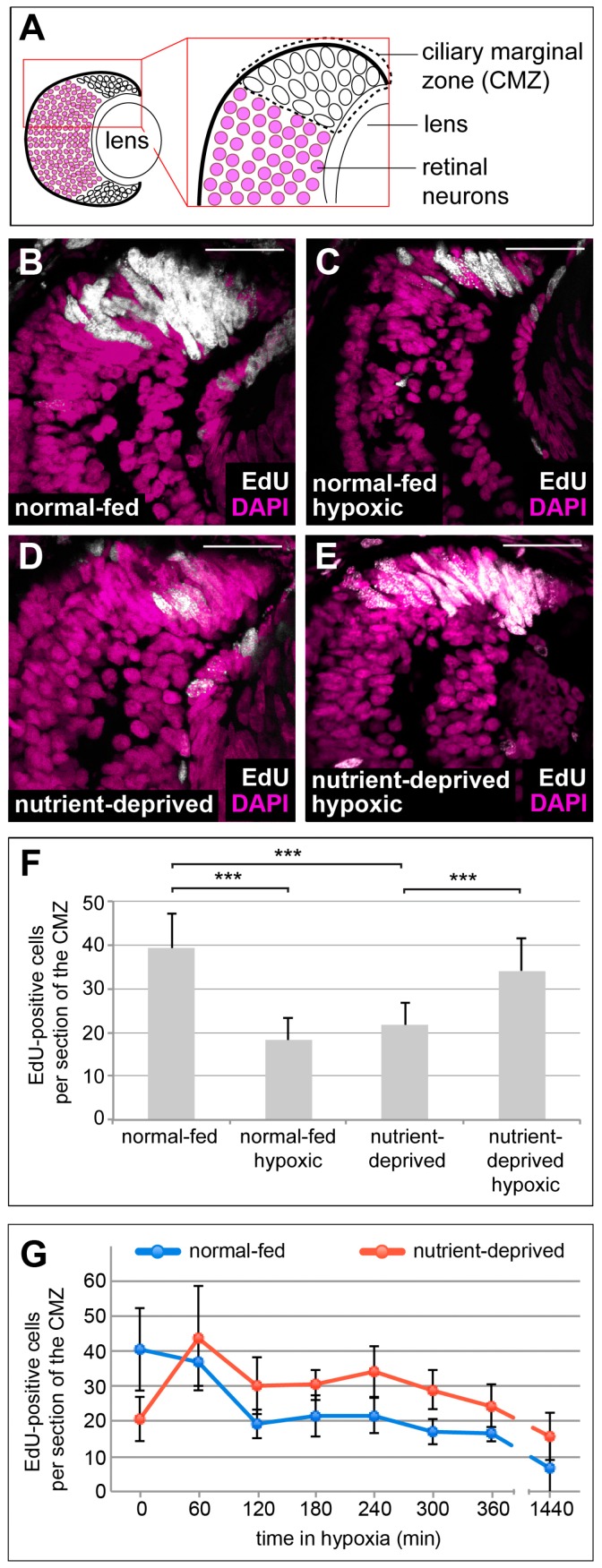
Hypoxia affects proliferation of progenitors in the retinal stem cell niche in the opposite manner, dependent on the nutritional status. (**A**) Schematic representation of a cross section through a 38-stage *Xenopus* retina, indicating the position of the ciliary marginal zone (CMZ). (**B**–**E**) EdU incorporation measured after a 2 h EdU pulse in DAPI-stained retinas from animals that have been normal-fed (**B**,**C**) or nutrient-deprived for 24 h (**D**,**E**) incubated under normoxia (**B**,**D**) or hypoxia (**C**,**E**). Nutrient deprivation or hypoxia reduced the number of proliferating cells in the CMZ, whereas hypoxic nutrient-deprived retinas show an increase of proliferation in the CMZ. Scale bars = 50 µm. For each condition, between 10 and 15 retinas were quantified. (**F**) Quantification of the experiment performed in (**B**–**E**). Error bars represent standard deviations. Two hooked ends of horizontal lines indicate the two conditions compared, *** *p*-value < 0.001; *n* = 7. (**G**) Quantification of EdU-positive cells after an 1 h EdU incorporation in retinas from normal-fed (blue line) or nutrient-deprived (red line) animals after various times in hypoxia (time in minutes indicated on the x-axis). Error bars represent standard deviations of two independent experiments (*n* = 2). For each condition and time point, a minimum of 10 retinas was quantified.

To determine whether hypoxia affects proliferation in the CMZ, we incubated embryos in a hypoxic chamber maintained at 5% oxygen and monitored proliferation by incorporation of the nucleoside analogue of thymidine, EdU. Embryos were placed in a 2 mM solution of EdU for 2 h and proliferative cells were labelled by incorporation of EdU into DNA during active DNA synthesis. As expected, normoxic control retinas showed intense EdU staining in the CMZ, where proliferating progenitors reside (Figure 1A,B,F). After oxygen deprivation, a strong decrease in CMZ progenitor proliferation was observed (Figure 1C,F). 

Having previously shown that CMZ cells are facultatively glycolytic, *i.e.*, they show Warburg metabolism in normoxia and sustained levels of ATP production when exposed to hypoxia [6], we wondered whether these cells might also be resistant to hypoxia with respect to proliferation. We also wondered whether the response to hypoxia on cell proliferation might depend on cellular context, particularly access to nutrients, similar to cancer cells that often proliferate in poorly vascularized niches with poor nutrient and oxygen availability [7,8,30]. To test how nutrient deprivation affects the proliferative response of CMZ cells to hypoxia, we repeated the EdU incorporation experiment using ND embryos in the hypoxic chamber. Strikingly, we found that in the CMZ of ND animals, proliferation was upregulated upon exposure to hypoxia (Figure 1B–F). This increase in proliferation was acute and greatest within 1 h, but was maintained above that found in ND-alone CMZs for many hours (Figure 1G). This result suggests that there are opposing effects of hypoxia on proliferation in nutrient replete *versus* starved condition. 

### 3.2. Activity of HIF-1 Signalling Regulates Proliferation of ND Retinal Progenitors

**Figure 2:**
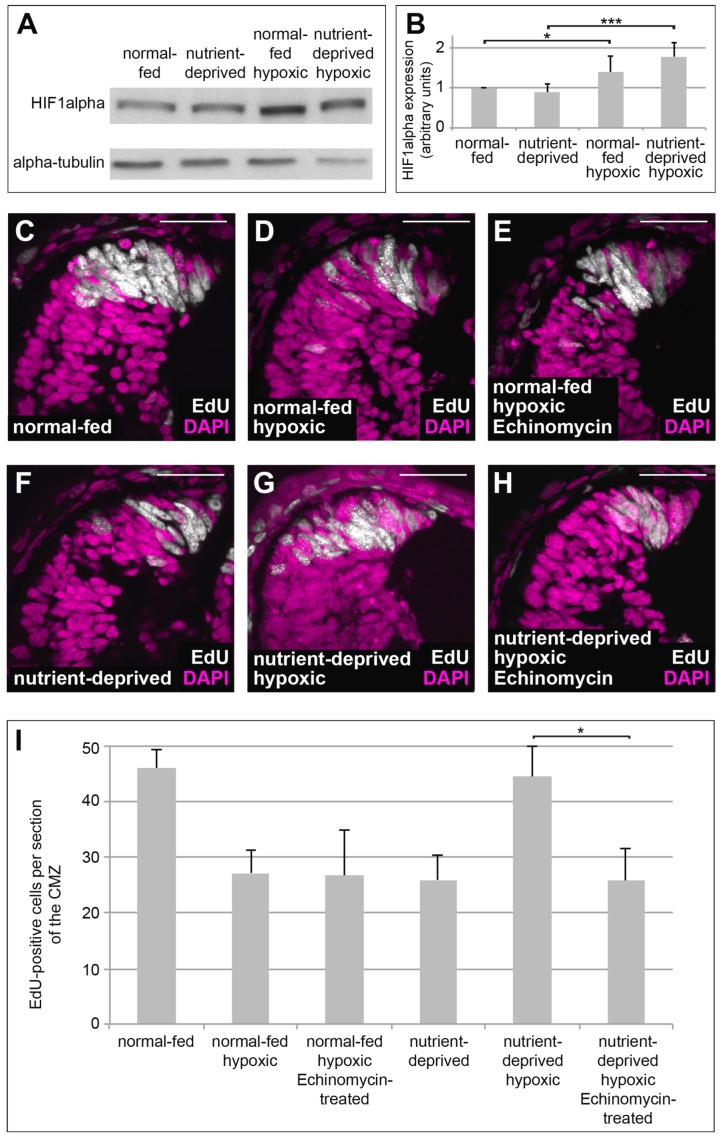
HIF-1 activity is required to induce re-initiation of proliferation in nutrient-deprived hypoxic retinas. (**A**) Western blot of retinas isolated from normal-fed or nutrient-deprived animals kept under normal oxygen concentrations or in hypoxia, probed for HIF-1α subunit and α-tubulin. HIF-1α is stabilized under hypoxia in both normal-fed and nutrient-deprived animals. At least 22 retinas were taken for each condition; all conditions in each experiment have the same number of retinas (*n* = 5). (**B**) Quantification of the Western blot performed in (**A**). Error bars represent standard deviations. Two hooked ends of horizontal lines indicate the two conditions compared, * *p*-value < 0.05, *** *p*-value < 0.001; *n* = 5. (**C**–**H**) EdU incorporation in DAPI-stained retinas from normal-fed animals (**C**–**E**) kept in normoxia (**C**), hypoxia (**D**) or incubated with Echinomycin under hypoxia (**E**) and in nutrient-deprived animals kept in normoxia (**F**), hypoxia (**G**) or incubated with Echinomycin under hypoxia (**H**). Nutrient-deprived retinas no longer resume their proliferation in hypoxia when function of HIF-1 is blocked by Echinomycin. Scale bars = 50 µm. In each condition, a minimum of 10 retinas was examined. (**I**) Quantification of the experiment performed in (**C**–**H**). Error bars represent standard deviations between three independent experiments. Two hooked ends of horizontal lines indicate the two conditions compared, * *p*-value < 0.05; *n* > 3. For each condition, between 10 and 15 retinas were quantified.

One of the major regulators of cellular metabolism and proliferation under hypoxia is HIF-1, the α subunit of which is sensitive to oxygen concentration [31]. In proliferating CMZ cells of the *Xenopus* retina, HIF-1α is stabilized under hypoxia in both normal-fed and ND retinas (Figure 2A,B). To investigate, whether re-initiation of proliferation in hypoxic nutrient-deprived retinas depends on the function of HIF-1, we blocked HIF-1 transcriptional activity by treatment with the specific HIF-1-inhibitor Echinomycin [32] and monitored EdU incorporation into the CMZ. While treatment with Echinomycin did not rescue reduction of proliferation in normal-fed hypoxic samples (Figure 2C–E,I), it entirely prevented the increase in proliferation seen in the CMZs of ND retinas (Figure 2F–H,I), which was not due to increased apoptosis (Figure S1A,B,D,E,G). This result suggests that the increase of proliferation observed in ND retinas following hypoxia seems to rely on HIF-1 activity.

### 3.3. Glutaminolysis is Critical for Proliferation in Response to Hypoxia in ND Retinal Progenitors

**Figure 3:**
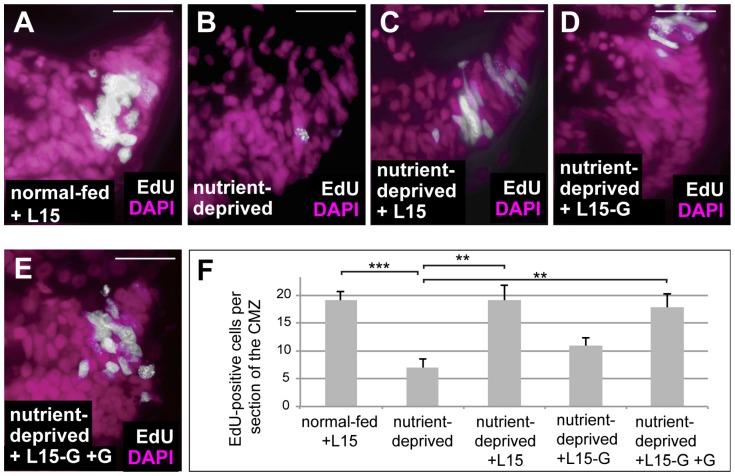
Glutamine is essential for retinal progenitor cell proliferation. (**A**–**E**) EdU incorporation in DAPI-stained *ex vivo* retinal explants from normal-fed embryos maintained in L15 medium for 24 h (**A**), from ND embryos maintained in 1× MBS for 24 h (**B**) or from ND embryos re-fed for 24 h with L15 (**C**), L15 lacking glutamine (**D**) or L15 lacking glutamine with added glutamine (**E**). Removal of glutamine from L15 medium prevented the rescue in proliferation observed following re-feeding. (**F**) Quantification of the experiment performed in (**A**–**F**). Error bars are standard deviations between three independent experiments. Two hooked ends of horizontal lines indicate the two conditions compared, ** *p-*values < 0.01, *** *p-*value < 0.001; scale bars = 20 μm; *n* = 3. Each experiment used six retinas per condition. These were from six different animals per condition (*i.e.*, one retina from each animal).

**Figure 4:**
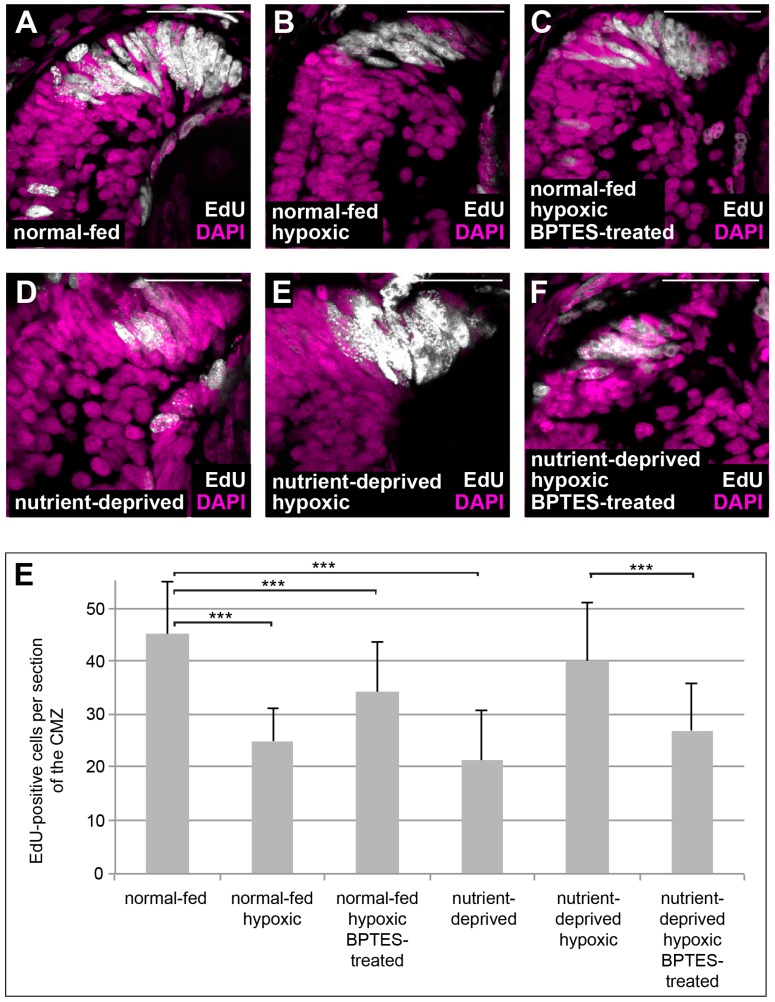
Block in glutaminolysis interferes with re-initiation of proliferation in nutrient-deprived hypoxic retinas. (**A**–**F**) EdU incorporation in DAPI-stained retinas from normal-fed animals (**A**–**C**) kept in normoxia (**A**), hypoxia (**B**) or incubated with BPTES under hypoxia (**C**) and in nutrient-deprived animals kept in normoxia (**D**), hypoxia (**E**) or incubated with BPTES under hypoxia (**F**). Nutrient-deprived retinas no longer resume their proliferation in hypoxia when glutaminolysis is blocked by BPTES. Scale bars = 50 µm. In each condition, a minimum of 8 retinas was observed. (**E**) Quantification of the experiment performed in (**A**–**F**). Error bars represent standard deviations between three independent experiments. Two hooked ends of horizontal lines indicate the two conditions compared, *** *p*-value < 0.001, *n* = 3. For each condition, between 8 and 17 retinas were quantified.

One of the major effects of HIF-1 is the rewiring of cellular metabolism towards activation of glycolysis and glutaminolysis [33]. Glutaminolysis involves the deamination of glutamine and subsequent generation of α-ketoglutarate, which serves to replenish the TCA cycle [34,35]. Retinas from ND animals can be re-fed in an *ex vivo* explant culture system using L15 medium [5]. We re-fed ND retinal explants using L15 medium both with and without glutamine and found that proliferation was only rescued when glutamine was present in the culture medium (Figure 3), suggesting that glutamine is essential for proliferation of *Xenopus* CMZ. Glutaminolysis can be blocked by BPTES, a specific inhibitor of the key enzyme glutaminase. To investigate whether glutaminolysis is necessary for the proliferative response of ND CMZ cells exposed to low oxygen, we treated normal-fed and ND embryos under hypoxia with BPTES and monitored proliferation in the CMZ by EdU incorporation. Our results show that treatment with BPTES interferes with upregulation of proliferation in the CMZs of ND animals placed in hypoxia (Figure 4), which was not due to increased apoptosis (Figure S1A–C,D–F). Thus, glutaminolysis, presumably upregulated by HIF-1, is essential for the increase in proliferation induced by hypoxia in ND conditions.

### 3.4. Increased Proliferation in Hypoxic ND Retinas Requires Reactivation of mTOR Signalling

**Figure 5:**
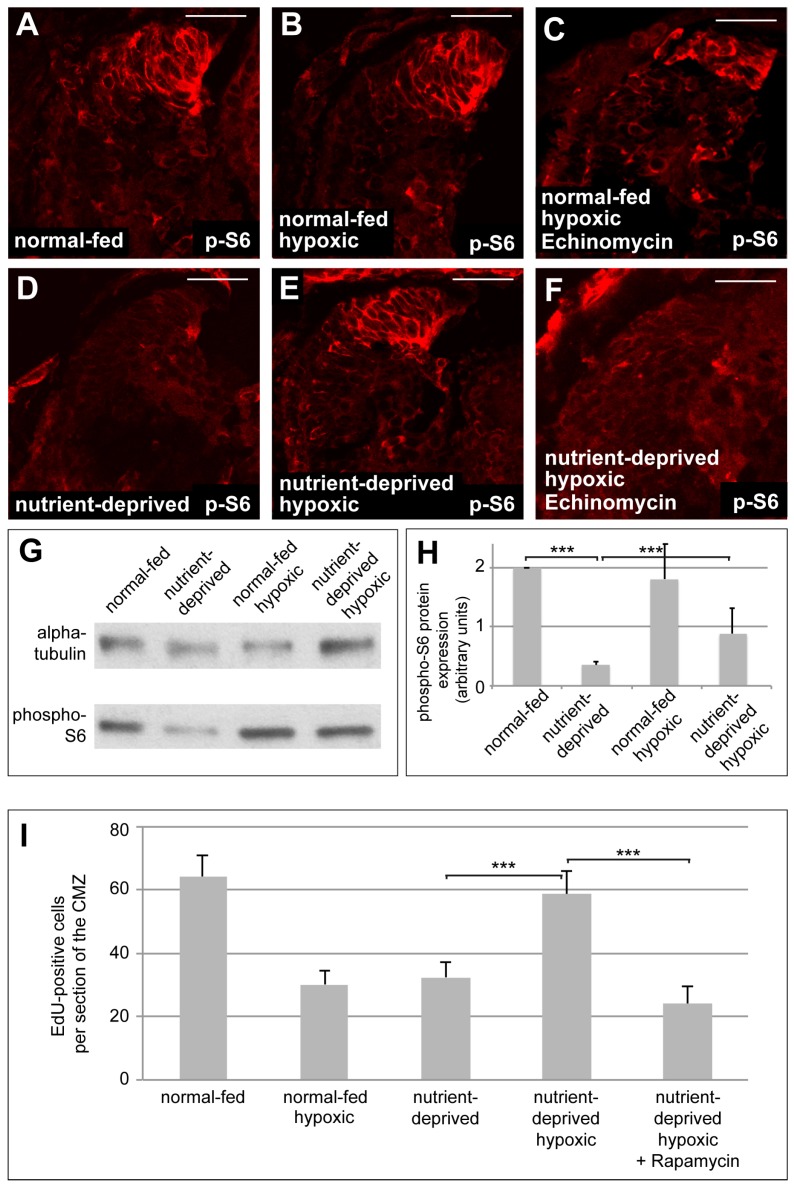
Reactivation of mTOR signalling, mediated by HIF-1 function, is required when nutrient-deprived retinas increase proliferation in hypoxia. (**A**–**D**) Immunostaining for the mTOR target phospho-S6 (p-S6) in retinas from normal-fed (**A**–**C**) or nutrient-deprived (**C**–**E**) animals kept in normal oxygen concentration (**A**,**D**), under hypoxia (**B**,**E**) or under hypoxia and treated with Echinomycin (**C**,**F**). Incubation of nutrient-deprived retinas in hypoxia restores p-S6 staining in the CMZ. Echinomycin treatment interferes with the increase of p-S6 staining in the CMZ of nutrient-deprived hypoxic retinas. Scale bars = 50 µm; *n* = 3. In each condition, a minimum of 8 retinas was examined. (**G**) Western blot of retinas dissected from normal-fed or nutrient-deprived normoxic or hypoxic animals, probed for phospho-S6 and α-tubulin. At least 22 retinas were taken for each condition, and all conditions in each experiment have the same number of retinas (*n* = 10). (**H**) Quantification of the Western blot shown in (**G**). Phospho-S6 levels, normalized to tubulin, are significantly reduced upon nutrient deprivation, unaffected by hypoxia alone, and increase to 40% of control levels upon nutrient deprivation in hypoxia. In Western blot quantification, error bars represent standard deviations between 10 independent experiments. Two hooked ends of horizontal lines indicate the two conditions compared, *** *p*-values < 0.001; *n* = 10. (**I**) Quantification of EdU incorporation in retinas from normal-fed or nutrient-deprived animals kept in normoxia, hypoxia or incubated with Rapamycin under hypoxia. Nutrient-deprived retinas no longer resume their proliferation in hypoxia when mTOR signalling is blocked by Rapamycin. Error bars represent standard deviations between two independent experiments. Two hooked ends of horizontal lines indicate the two conditions compared, *** *p*-values < 0.001; *n* = 2. For each condition, between 7 and 15 retinas were quantified.

Recent studies show that glutaminolysis can activate mTOR signalling [36]. As mTOR is closely involved in regulating proliferation in response to nutritional levels in *Xenopus* CMZ tissue [5], we hypothesized that the increased proliferation in the ND CMZ may be due to the effects of glutaminolysis on mTOR signalling. We measured the levels of phospho-S6 kinase (pS6), a target of activated mTOR signalling, by Western blot and immunostaining, in normal-fed and ND retinas under normoxia and hypoxia. As expected, normoxic ND retinas showed decreased pS6 staining in the CMZ, as well as lower levels of pS6 protein, compared to normal-fed retinas (Figure 5A,D,G,H and [5]). pS6 staining, however, remained high in the CMZ of normal-fed retinas upon induction of hypoxia (Figure 5B). In contrast, ND CMZs showed an increase of pS6 protein levels, as well as pS6 staining intensity in the CMZ when exposed to hypoxia (Figure 5E,G,H). Treatment with Echinomycin, a small molecule inhibitor of HIF-1 activity, interfered with this upregulation of pS6 staining in hypoxic ND CMZs, but not in normal-fed CMZs, implicating HIF-1 as critical in this process (Figure 5C,F).

Taken together, these results suggest that mTOR signalling may be required for reactivation of proliferation when starved CMZs are exposed to hypoxia. To further test this hypothesis, we monitored proliferation in ND CMZs exposed to hypoxia when the mTOR pathway was blocked by the addition of the mTOR inhibitor Rapamycin. Indeed, as expected, we found ND retinal progenitors could no longer reinitiate their proliferation in hypoxia when incubated in Rapamycin (Figure 5I). This suggests that under hypoxic conditions HIF-1 activates glutaminolysis, leading to the derepression of the mTOR pathway in nutrient-deprived proliferating cells and this accounts for the increase of their proliferation upon exposure to hypoxia.

## 4. Discussion

It seems sensible that starving cells should strive to decrease their metabolic activity, limit proliferation, and activate mechanisms essential for cell survival. We previously showed that the mTOR pathway is the main regulator of proliferation in response to starvation in the neurogenic CMZ of *Xenopus* [5]. Interestingly, we now show that the low rate proliferation in ND retinal progenitors *in vivo* is increased when these cells also become hypoxic. The finding is counterintuitive and suggests that ND progenitors have a different metabolic state than nourished ones. Unfortunately, the limitations of the *Xenopus* embryo system make it challenging to measure the specific metabolic profiles of CMZ cells. Our experiments suggest, however, that the effect involves HIF-1 signalling, which normally activates glycolysis and glutaminolysis. Both HIF-1 activity and glutaminolysis are essential for the increased proliferation in ND retinas exposed to hypoxia. Interestingly, cancer cells upregulate HIF-1 signalling and depend strongly on glutaminolysis for energy [16,37]. The response of normal proliferating cells to the conditions that cancer cells often experience suggests that it may be a natural adaptation for such cells to increase proliferative activity when confronted with both ND and hypoxia. 

We show that hypoxia induces an upregulation of mTOR activity in ND CMZ cells. A similar mechanism has been recently proposed for umbilical cord blood-derived human mesenchymal stem cells (UCB-hMSCs), where hypoxia induces transient proliferation via upregulation of mTOR signalling [38]. It has also been shown that hypoxia-induced HIF-1 signalling activates fatty acid synthase to synthesize lipids [38] and that HIF-2 affects lipid handling in mouse retinal pigment epithelium cells [39]. Our findings in the CMZ are also in line with those of [24] who found that smooth muscle endothelial cells *in vitro* are induced to proliferate in hypoxia via a HIF-1-mediated increase in mTOR signalling. 

Proliferating cells in the retina use a Warburg metabolism to fuel anabolic substrates for growth. This reduces the dependence on oxygen, but hypoxia often leads to the uncoupling of the electron transport chain, which can lead to the production of reactive oxygen species (ROS) [40,41]. ROS production regulates various signalling pathways related to proliferation and differentiation [42,43,44,45,46,47] and aids in tissue regeneration in *Xenopus* [48], yet ROS are known to be toxic [41]. Interestingly, a recent study shows that, in response to ND, lipid droplets that serve as ROS scavengers form in the glia cells surrounding the neuroblast niche, thereby protecting proliferating progenitors from ROS damage [49]. We have not investigated the role of ROS in the CMZ but it would be interesting to know what role ROS might play in the integration of the HIF-1 and mTOR pathways that affect proliferation. It is possible that HIF-1-mediated induction of glutaminolysis in hypoxia may also serve to limit ROS production through replenishment of glutathione, a major cellular ROS quencher [50,51]. Similar mechanisms may be at play in proliferative cancer cells that rely strongly on glutamine supply, but often display a high production of ROS to support proliferation [44,45,50,52,53]. 

Taken together, our study suggests that non-transformed cells *in vivo* during normal development may be naturally programmed to increase their proliferation when they are facing both nutrient and oxygen deprivation. In our experiments, a combination of ND and hypoxia has a beneficial effect on retinal proliferation, albeit for a limited period of time. This is an interesting finding, as ND hypoxic retinas show a reactivation of mTOR pathway, while mTOR upregulation is generally associated with increase in cellular senescence, and hypoxia suppresses geroconversion [54]. However, the relationship between aging, hypoxia and mTOR pathway is complex and additional studies are necessary to determine the specific interactions between HIF-1 and mTOR in non-transformed and cancer cells. While hypoxia may be a common feature of many solid tumours, cancer cells can react differently to chemotherapeutic drugs that influence different aspects of cellular metabolism, as a recent study by Strese and colleagues has shown [55]. Interestingly, their results also show that cultured cancer cells from solid tumours, such as ovarian and small lung cancer cells, were more sensitive to Rapamycin when treated under hypoxic conditions, suggesting that proliferation of transformed cells might be strongly supported by mTOR pathway in hypoxia. Whether this is adaptive response of normal proliferating cells or a maladaptive response that cancer cells in solid tumours take advantage of, remains an important but open question.

## Supplementary Materials

The following are available online at www.mdpi.com/2221-3759/4/2/17/s1.

## Abbreviations

The following abbreviations are used in this manuscript:

ND: nutrient-deprived; nutrient deprivation
CMZ: ciliary marginal zone
mTOR: mechanistic Target of Rapamycin
HIF-1: Hypoxia-Inducible Factor 1
MBS: Modified Barth’s solution
BPTES: bis-2-(5-phenylacetamido-1,3,4-thiadiazol-2-yl)ethyl sulfide
EdU: 5-ethynyl-2′-deoxyuridine
PSF: Penicillin/ Streptomycin/ Amphotericin
L-15: Leibovitz medium
DAPI: 4′,6-diamino-2-phenylindilone
RIPA buffer: radioimmunoprecipitation assay buffer
SDS: sodium dodecyl sulfate
HRP: horseradish peroxidase
HIF-1α: Hypoxia-Inducible Factor 1α
TCA: tricarboxylic acid
G: Glutamine
pS6: phospho-S6 kinase
ROS: reactive oxygen species
UCB-hMSCs: umbilical cord blood-derived human mesenchymal stem cells
