# Supplementary material for: Nutrient-Deprived Retinal Progenitors Proliferate in Response to Hypoxia: Interaction of the HIF-1 and mTOR Pathway
Source: J Dev Biol. 2016 May 19;4(2):17. doi: 10.3390/jdb4020017 (PMC4894462; doi:10.3390/jdb4020017)
Supplement: Supplementary file 1 [file jdb-04-00017-s001.pdf]

Supplementary Materials: Nutrient-Deprived Retinal Progenitors Proliferate in Response to Hypoxia: Interaction of the HIF-1 and mTOR Pathway

Helena Khaliullina *, Nicola K. Love and William A. Harris *

Supplementary Methods:

Normal-fed and nutrient-deprived embryos at stage 38 were placed into a hypoxic bath chamber maintained under a constant infusion of a mixture of 5% oxygen and 95% CO_2. These embryos were incubated in the hypoxic bath chamber for 5 h and were either untreated or treated with 100 nM Echinomycin (Sigma-Aldrich, Gillingham, UK) or 50 μM BPTES (Sigma-Aldrich). The embryos were then fixed, sectioned and the sections were treated with the InSitu Cell Death Detection Kit Fluorescein (Sigma-Aldrich) according to the manufacturers instructions and viewed and recorded under the fluorescent microscope. The positive control was treated with DNAse, which resulted in DNA fragmentation that is detected with the kit. The *In Situ* Cell Death Detection Kit Fluorescein is based on the detection of single- and double-stranded DNA breaks that occur at the early stages of apoptosis.

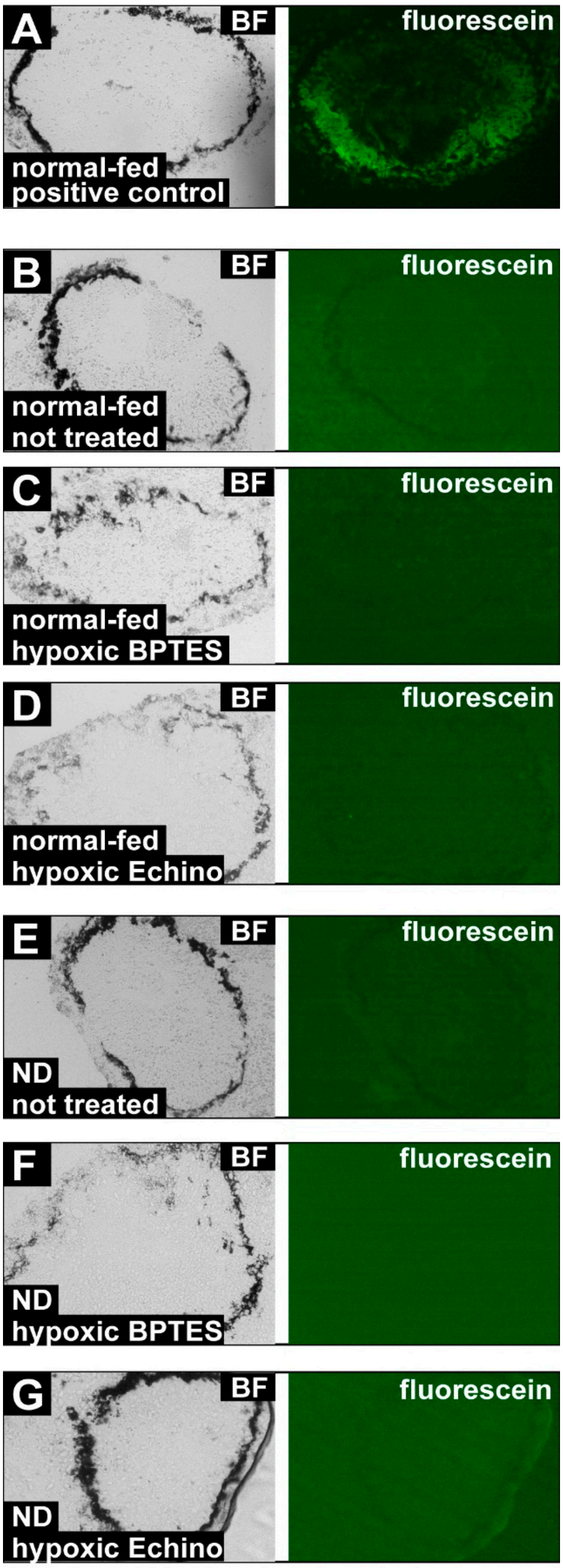

Figure S1. BPTES- or Echinomycin-treated normal-fed or nutrient-deprived hypoxic retinas do not exhibit increased apoptosis. (**A**–**G**) Bright-field (**B**,**F**) or fluorescein images of retinal cross-sections from normal-fed (**A**–**D**) or nutrient-deprived (**E**–**G**) embryos treated as described. (**A**) The positive control retinal section from a normal-fed untreated embryo that has been incubated with DNAse. This retinal section shows clear fluorescein signal, confirming that DNA fragmentation is detected in this assay. (**B**–**G**) No positive fluorescein signal, indicating that no DNA fragmentation has resulted from the indicated treatments (n = 2).
